# Cancer neoantigens as potential targets for immunotherapy

**DOI:** 10.1007/s10585-021-10091-1

**Published:** 2021-05-05

**Authors:** Weijie Ma, Brian Pham, Tianhong Li

**Affiliations:** 1 Division of Hematology/Oncology, Department of Internal Medicine, University of California Davis School of Medicine, University of California Davis Comprehensive Cancer Center, 4501 X Street, Suite 3016, Sacramento, CA 95817 USA; 2 Medical Service, Hematology and Oncology, Veterans Affairs Northern California Health Care System, Mather, CA USA

**Keywords:** (4–6) Cancer neoantigen, Tumor mutational burden, Cancer vaccine, Tumor genomic profiling, Personalized immunotherapy

## Abstract

Immune checkpoint inhibitors (ICIs) targeting the cytotoxic T-lymphocyte-associated protein-4 (CTLA-4) and programed cell death protein 1 (PD-1) or its ligand PD-L1 have increased the survival and cure rates for patients with many cancer types in various disease settings. However, only 10–40% of cancer patients benefited from these ICIs, of whom ~ 20% have treatment interruption or discontinuation due to immune-related adverse events that can be severe and even fatal. Current efforts in precision immunotherapy are focused on improving biomarker-based patient selection for currently available ICIs and exploring rationale combination and novel strategies to expand the benefit of immunotherapy to more cancer patients. Neoantigens arise from ~ 10% of the non-synonymous somatic mutations in cancer cells, are important targets of T cell-mediated anti-tumor immunity for individual patients. Advances in next generation sequencing technology and computational bioinformatics have enable the identification of genomic alterations, putative neoantigens, and gene expression profiling in individual tumors for personal oncology in a rapid and cost-effective way. Among the genomic biomarkers, defective mismatch DNA repair (dMMR), microsatellite instability high (MSI-H) and high tumor mutational burden (H-TMB) have received FDA approvals for selecting patients for ICI treatment. All these biomarkers measure high neoantigen load and tumor antigenicity, supporting the current development of neoantigen-based personalized cancer vaccines for patients with high TMB tumor. Several studies have shown neoantigen vaccines are feasible, safe and have promising clinical activity in patients with high TMB tumors in both metastatic and adjuvant settings. This review summarizes the emerging data and technologies for neoantigen-based personalized immunotherapy.

## Introduction

Cancer immunotherapy refers to a diverse range of therapeutic approaches that aim to harness the immune system to establish targeted antitumor immune responses [1, 2]. Cancer cells have cumulative, nonsynchronous somatic mutations, which make them potentially antigenic and recognizable by the immune system. However, cancer cells can evade the immune surveillance by several mechanisms such as disruption of antigen presentation, modulation of checkpoint pathways, tumor infiltration of immunosuppressive cells, and upregulation and secretion of immunosuppressive cytokines [3]. The field of cancer immunotherapy has undergone a renaissance due to a better understanding of the complex pathways that regulate tumor-induced immunosuppression [4]. First-generation immune checkpoint inhibitors (ICIs) targeting cytotoxic T-lymphocyte-associated protein-4 (CTLA-4) and programed cell death protein 1 (PD-1) or its ligand PD-L1 have become the most potent and durable cancer immunotherapy for patients with many cancer types in various disease settings [5]. Currently, FDA-approved ICIs include the anti-PD-1 monoclonal antibodies (mAbs) nivolumab and pembrolizumab; the anti-PD-L1 mAbs atezolizumab, durvalumab, avelumab, and cemiplimab; and the anti-CTLA-4 mAb ipilimumab [6–10]. These ICIs have improved the overall survival in patients with many cancer types in various disease settings. Using lung cancer as an example, ICIs have been approved as first-line, second-line or consolidation treatment for patients with non-small cell lung cancer (NSCLC), first-line therapy for patients with metastatic small cell lung cancer (SCLC) [11], melanoma [12] and unresectable malignant pleural mesothelioma [13]. However, the clinical benefit of ICIs is quite variable among different solid tumor types and objective tumor responses and durable long-term disease control are seen in only 10–40% of unselected patients with these solid tumor types [14]. High PD-L1 expression on the membrane of tumor cells by immunohistochemistry (IHC), defective mismatch DNA repair (dMMR), microsatellite instability high (MSI-H) and high tumor mutational burden (TMB-H) have been approved as companion diagnostics for PD-1 inhibitor pembrolizumab for selected or pan-tumor types [15, 16] (Table 1). Compared to molecular biomarkers, such as *gain-of-function EGFR* mutations and *ALK* gene rearrangements, which predict 60–80% of clinical responses to molecularly targeted therapy in NSCLC, immune biomarkers predict up to 50% with significant variations among different ICIs and tumor types [17]. Furthermore, cancer patients receiving ICIs may develop unique (and in rare cases, fatal) immune-related adverse events (irAEs) that can affect any organ due to inflammatory infiltration of activated immune cells attacking normal organs [18–20]. Ongoing effects aim to develop and validate minimally invasive immune-oncology biomarker assays that select the appropriate patients for cancer immunotherapy and monitor treatment response. There have also been extensive efforts to understand the resistance to ICIs, explore rationale combination, and to develop new strategies to improve the efficacy and reduce the off-target adverse effects of cancer immunotherapy that have been elegantly reviewed elsewhere [15, 21]. In this review, we will summarize the emerging data and technologies especially for using cancer neoantigens as potential targets for personalized immunotherapy.

**Table 1:** Summary of genomic biomarkers for ICIs

Biomarker	Diagnostics	Tumor Types	Agents
dMMR	Companion	Pan tumor types	Pembrolizumab
MSI-H	Companion	Pan tumor types	Pembrolizumab
TMB	Companion	Pan tumor types	Pembrolizumab
bTMB	Companion (pending)	Selected tumor types	Atezolizumab
GEP	Under development	Pan tumor types	Pembrolizumab, nivolumab

*dMMR* deficient mismatch repair; *TMB* tumor mutation burden; *MSI-H* microsatellite instability high; *GEP* gene expression profiling

## Cancer immunity and tumor microenvironment

As reviewed previously [16], the tumor microenvironment (TME) includes tumor cells and its surrounding blood vessels, fibroblasts, immune cells (e.g., lymphocytes), bone marrow-derived suppressed cells, extracellular matrix (ECM), and signalling molecules (e.g., interleukin (IL)-1, interferon-gamma (IFN-γ). Tumors can influence the microenvironment by releasing extracellular signals and stimulating peripheral immune tolerance, while the immune cells in the microenvironment can affect the growth, proliferation, and evolution of cancer cells [22]. In the TME, a series of stepwise events are initiated in the cancer-immunity cycle that lead to effective killing of cancer cells. There are two major phases. First, initiation of antitumor immunity begins with antigen release, antigen capture, processing by dendritic cells, release of immunogenic signals, and T-cell priming. Second, cancer-specific cytotoxic T lymphocytes (CTLs) is activated, resulting in the trafficking of CTLs into the TME and the killing of cancer cells through the interaction between the T cell receptor (TCR) and its cognate antigen bound to major histocompatibility complex (MHC)-I on antigen-presenting cells (APCs) [5]. Cytotoxic and helper T cells play essential roles in killing the cancer cells and long-term tumor control. The killing of tumor cells results in the release of additional neoantigens and tumor-associated antigens (TAAs). Cancer cells also have the ability to upregulate PD-L1 expression to “turn off” effector CD8^+^ T cells, thus evading immune-mediated tissue destruction. This is the rationale for the development of novel immunotherapies that increase the numbers of effector CD8^+^ T cells and target immune checkpoints responsible for normalizing, re-establishing or augmenting effector CD8^+^ T cell function against tumor cell [23]. However, various immune effector cells that are recruited and interacted with tumor cells are downregulated in response to tumor-derived signals. Meanwhile, activation of molecular mechanism that leads to apoptosis of antitumor effector cells also contributes to tumor escape [24]. Based on the infiltration of inflammatory cells, TME has been stratified into either “hot” or “cold” tumors. “Hot” tumors comprise disease with a pro-inflammatory TME and tumor-infiltrating lymphocytes (TILs). On the contrary, “cold” tumors lack this inflammatory signature [25]. These immunologically hot tumors have several distinct features: higher gene expressions involved in activation of stimulator of interferon gene (STING) pathways, CD8^+^ TILs, T-cell recruiting chemokines, and dendritic cells [26]. These same tumors tend to have higher regulatory markers including PD-L1, IDO, Tregs, which counteract the pro-inflammatory features to promote TIL anergy and ultimately tumor immune evasion [26, 27]. Furthermore, activated T-cells self-regulate their activity and proliferation via so-called “exhaustion” markers such as lymphocyte-activation protein 3 (LAG3), T cell immunoglobulin and mucin domain-3 (TIM3), PD-1, and T-cell immunoreceptor with Ig and ITIM domains (TIGIT) to self-regulate proliferation and activation [28]. This may explain the responses seen using immunotherapy for such immunologically active tumors. In addition to the cell-mediated distant metastasis, exosome-mediated metastasis has been recognized as an independent mechanism, and recently has been found to regulate cancer immunity and responses to ICIs [29]. This knowledge of exosome-mediated metastasis and cancer immunity is important for developing therapeutic strategies to eliminate metastasis and biomarker to monitor tumor and immune responses. The IFN-γ pathway plays a key role in adaptive and acquired resistance to ICIs. Produced by tumor-specific T-cells, IFN-γ induces an effective antitumor immune response through the increasing presentation of tumor immunogenic proteins or facilitating a pro-apoptotic effect on tumor cells [30]. However, immune escape occurs from continuous IFN-γ exposure due to mutation or downregulation of IFN-γ signalling pathways including Janus kinase (JAK)1, JAK2, and the signal transducers and activators of transcription (STATs) [31]. Preclinical data has demonstrated resistance to PD-1 blockade immunotherapy was associated with defects in the IFN-receptor signalling and antigen presentation pathways [32]. JAK1 and JAK2 mutations have resulted in a lack of response to IFN-γ, causing resistance to immune therapy and cancer cell escape [33]. Copy number alterations in IFN-γ pathway genes for IFN-γ receptor 1 and 2 (IFNGR1 and IFNGR2), JAK2, and IFN regulatory factor 1 (IRF1) have been seen in tumor samples resistant to ipilimumab [34].

## Biomarkers for ICIs: tumor mutation burden (TMB) as a potential measurement for cancer neoantigens

**Fig. 1:**
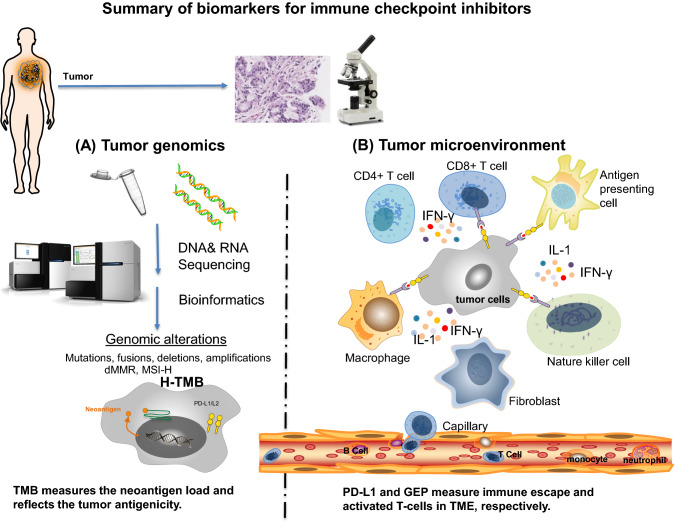
Summary of biomarkers for immune checkpoint inhibitors. The tumor microenvironment can be examined via histopathology and molecular studies. The relationship between tumor and immune cells can be evaluated by immunohistochemistry and molecular or genetic profiling analysis. Known biomarkers for immune checkpoint inhibitors characterize the properties of either tumor genomics (**a**) or tumor microenvironment (**b**). **a** Next generation sequencing (NGS) and bioinformatics identify genomic alterations in tumor cells, which include somatic mutations, fusions, deletions, amplifications, dMMR, MSI-H and H-TMB. Among these genomic biomarkers, TMB best measures the neoantigen load and reflects the tumor antigenicity. **b** TME includes tumor cells and its surrounding blood vessels, fibroblasts, immune cells (e.g., lymphocytes), bone marrow-derived suppressed cells, extracellular matrix (ECM), and signaling molecules (e.g., interleukin (IL)-1, interferon-gamma (IFN-γ). Tumor cells escape immune surveillance via PD-L1 expression. The combination of PD-L1 IHC and GEP better characterizes the immune escape and immune cell activity in TME. *dMMR* deficient mismatch repair; *H-TMB* high tumor mutation burden; *MSI-H* microsatellite instability high; *GEP* gene expression profiling; *PD-L1* programmed death-ligand 1; *IHC* immunohistochemistry

Tumor cells and immune cells in TME can be dissected histopathologically and molecularly to characterize spatial relationships between tumor and immune infiltrates by IHC, molecular or genetic profiling analysis, and cellular functional assays. Companion biomarkers for pembrolizumab monotherapy include high PD-L1 expression on the membrane of tumor cells alone or in combination of the membrane expression on immune cells by immunohistochemistry for selected cancer types, including NSCLC (2015), gastric cancer (2017), cervical (2018), urothelial cancer (2018), esophageal squamous cell carcinoma (2019) and head or neck squamous cell cancer (2019) [35–37]. Several genetic biomarkers, including dMMR, MSI-H and H-TMB, have been approved as companion diagnostics for PD-1 inhibitor pembrolizumab for pan-tumor types. Figure 1 summarizes these biomarkers for ICIs in the content of tumor cells and TME.

Figure 1a illustrates the key genomic biomarkers. Increased tumor somatic mutations likely form more neoantigens and TMB can represent a useful estimation of tumor nonantigenic load [38]. Thus, TMB can be a surrogate for measuring tumor antigenicity. dMMR and MSI-H are distinct genetic alterations leading to high TMB. MMR has been identified as a predictive biomarker of response to pembrolizumab in patients with hereditary non-polyposis colorectal cancer. However, some patients with intact MMR systems or microsatellite-stable tumors could still benefit from treatment with ICIs [39]. MSI-H tumors can express high levels of multiple immune checkpoint molecules, such as PD-1, PD-L1, CTLA-4. Some cancer subtypes, such as Merkel-cell carcinoma, have a better tumor response than would be predicted by the TMB alone, possibly due to the presence of viral antigens on tumor cells [40]. TMB-H (≥ 10 mutations/megabase, mut/mb) was independently associated with improved objective response rate (ORR) and longer clinical benefit in patients with metastatic NSCLC [41]. In June 2020, the US FDA approved pembrolizumab for the treatment of multiple metastatic solid tumors with high tumor mutational burden based on the phase II KEYNOTE-158 trial (NCT02628067) [42]. Although TMB-H is associated with response to pembrolizumab monotherapy, it does not predict the response to immune chemotherapy combination [43]. Further study is required to harmonize the different assays and determine the optimal cutoff for TMB as a predictive biomarker.

As illustrated in Fig. 1b, the tumor expression of PD-L1 mediates the evasion of immune surveillance and the expression of PD-1 on tumor-infiltrating lymphocytes (TIL) or tumor cells suggests the presence of effector T cells in TME. Thus, the detection of both PD-L1-positive tumor cells and PD-1-positive TIL at TME by IHC is a favorable prognostic factor and the best predictive factor of clinical response to ICI therapy. Phenotypic analysis of various immune cells in the TME and blood is usually performed by flow cytometry. Gene expression profiling (GEP) and transcriptome expression of T cell activation and inflammatory changes have shown in identifying genetic signatures that may predict response to ICIs in a variety of solid malignancies. In a cancer cohort, copy number loss of tumor suppressor genes was indicative of downregulated immune pathway mRNA expression and poor response to ICIs. The presence of a T-cell inflamed or activated GEP in addition to PD-L1 IHC has improved the prediction of favorable clinical response to pembrolizumab [44]. Additionally, pre-treatment genetic profiling has also yielded certain groups of genes or GEP signatures that are involved in antigen presentation, TCR complex formation and activation, immune co-stimulatory activation, apoptosis, and checkpoint inhibition. Recently, gene expression profiling analyses have been used to elucidate and stratify spatially distinct tumor immune microenvironments and genetic evolution for triple-negative breast cancers [45] and NSCLC [46].

The interaction of known immune biomarkers in patients receiving pembrolizumab monotherapy across all cancer types was retrospectively analyzed. While TMB, PD-L1 IHC, and T cell-inflamed GEP each captures distinct features of antigenicity and T cell activation, each of them independently predicts response to pembrolizumab. Combining cancer neoantigen assessment by TMB with inflammatory biomarkers (PD-L1 IHC and GEP for core pathways) better delineate the complexed tumor-immune cell interplays in TME. The combined biomarker approach is prospectively evaluated in Keynote-495 trial (NCT03516981) for the selection of different pembrolizumab-based combination therapy in patients with treatment-naive, advanced NSCLC. Based on the results of the biomarker screening, patients will be assigned to 1–4 groups: TMB_low_GEP_low_, TMB_high_GEP_low_, TMB_low_GEP_high_, and TMB_high_GEP_high_. Within each group, patients will be randomly assigned to receive a combination treatment of pembrolizumab and MK-1308 (anti–CTLA-4), MK-4280 (anti–LAG-3), or lenvatinib (receptor tyrosine kinase inhibitor), with the randomization assignment adaptively modified based on interim efficacy analyses [47].

## Cancer neoantigens

Cancer development is a complex process. Changes at the genetic level lead to modified intracellular signaling which cause changes in cellular behavior and gives rise to cancerous tissue. Eventually, organs and the entire organism are affected [47]. The roots of tumor immunology can be traced back to over a hundred years ago, when it was first demonstrated that antibodies could be produced against tumors. This finding supported the concept that tumors were “foreign” to the body [48]. Many efforts have been investigated to identify tumor targets that could elicit tumor immunity. There are two major types of cancer antigens. Tumor-associated antigens (TAAs) which have a higher expression level on cancer cells than normal cells are relatively restricted to tumor cells [49]. Cancer neoantigens, or tumor-specific antigen (TSA**)** is the consequences of the genetic alterations accumulated by cancer cells during the cancer genesis or epigenetic process [50]. The tumor-specific neoantigens generated by somatic mutations can be recognized by T-cells and influence patient response to immunotherapy [51]. After transcription and translation, the peptide containing neoantigens are processed by the antigen-processing machinery and loaded on to major histocompatibility complex (MHC) for presentation on the cell surface. Compared with TAAs, cancer neoantigens which are not affected by central immunological tolerance, have stronger immunogenicity and higher affinity to MHC-II, which elicits strong tumor immunity [50]. Not all somatic mutations generate neoantigens and only 10% of the non-synonymous mutations in tumor cells can produce antigenic peptides. Immunogenic neoantigens should have the following properties. First, the somatic mutations should alter the protein expression; Second, neoantigens can be properly processed and loaded on to MHC complexes to be recognized by the TCR of responding T cells [51]. Third, each tumor has multiple yet unique clonal/trunk and subclonal neoantigens. High burden of clonal/trunk neoantigens but not subclonal neoantigens is associated with high risk of tumor recurrence and poor survival in patients with early stage NSCLC [52]. Next generation sequencing (NGS) technology and computational bioinformatics have helped to fingerprint the genetic makeups of individual tumors, identify neoantigen candidates, and profile immune systems for personal oncology in a rapid and cost-effective way.

T cells and T cell receptors (TCRs) play a critical role in adaptive immune responses against cancers. In the TME, T-cells generate a diverse TCR repertoire through somatic gene rearrangements to tumor antigens [53]. The diversity of TCR repertoire is higher in tumors than that in non-tumor tissues in several cancer types (lung cancer, breast cancer and colon cancer) and peripheral blood [54–57]. Given the landscape of neoantigens is heterogenous and unique for each tumor in individual patients, the clonicity and diversity of T cell repertoire to neoantigens is also unique in individual patients. As the tumor progresses, the amount and diversity of neoantigens also evolves [58, 59]. In addition, tumor killing also releases additional neoantigens and tumor-associated antigens (TAAs) [58]. The diversity of TCR repertoire increases during the evolution of tumor progression with increased neoantigens and TAAs in both TME and sentinel lymph nodes [54–56]. Patients with higher TCR diversity have improved clinical responses to ICIs than those with lower TCR diversity in lung and cervical cancer [56, 60, 61]. The clonality and diversity of T-cell repertoires to neoantigens vary in tumors, non-tumor tissues and peripheral blood [56, 60], which can evolve during cancer progression. Anti-tumor immune responses require the functional presentation of tumor antigens and a TME that is replete with competent immune effectors. Immune infiltration varies both between and within tumors, with different mechanisms of neoantigen presentation dysfunction enriched in distinct immune microenvironments. In a large-scale meta-analysis of over 1000 IPI-treated cases with exome/transcriptome data, clonal but not subclonal TMB was the strongest predictor of IPI response [62]. Immune infiltration varied both between and within tumors, with different mechanisms of neoantigen presentation dysfunction enriched in distinct immune microenvironments. Immune-infiltrated tumor regions exhibited ongoing immunoediting, with either loss of heterozygosity in human leukocyte antigens or promoter hypermethylation/depletion of expressed neoantigens. Thus, current neoantigen vaccines are designed to target multiple clonal neoantigens to optimize the cancer immunity for individual patients.

## Recent development of cancer vaccines using cancer neoantigens

**Fig. 2:**
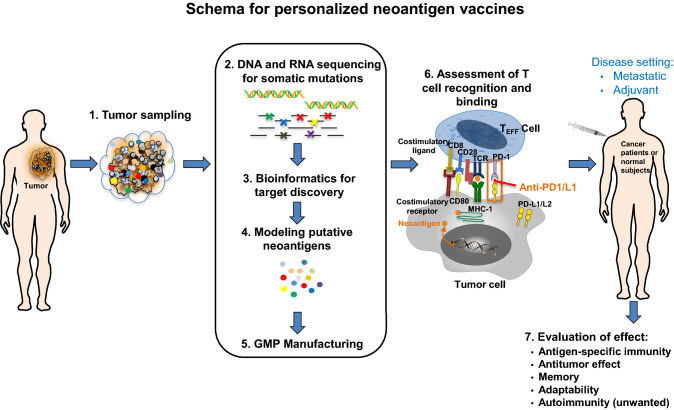
Schema for generating personalized neoantigen vaccines. Six key steps to manufacture personalized neoantigen vaccines include: (1) tumor sampling, (2) DNA and RNA sequencing for tumor-specific mutations, (3) bioinformatic analysis for target discovery, (4) in silico analysis for putative neoantigens, (5) neoantigen vaccine production under good manufacture practice (GMP), (6) assessment of T cell recognition and binding, and (7) evaluation of neoantigen vaccine effect in antigen-specific immunity, antitumor activity, memory, adaptability, and autoimmunity in different disease settings. *DNA* deoxyribonucleic acid; *RNA* ribonucleic acid

Cancer vaccines are designed to elicit the immune system's ability to recognize and kill cancer cells [63]. Major types of cancer vaccines include nucleic acids, dendritic cell (DC)-based tumor cell, and synthetic long peptide (SLP) vaccines. In 2010, the FDA has approved Provenge (dendritic cells expression of tumor antigen prostatic acid phosphatase) for men with metastatic prostate cancer. Peptides vaccines have been the main target of cancer vaccines and they have been proved well tolerated. Recent knowledge on tumor immunology and advances on bioinformatics technology on identification and production of putative cancer neoantigens enable the conduction of clinical trials using personalized therapeutic cancer vaccines. Figure 2 summarizes the key steps generating neoantigen-based cancer vaccine. It is crucial to select the cancer neoantigen targets among the diverse, putative cancer neoantigen targets for individual tumors. Due to the complex immune tolerance mechanisms, cancer vaccines alone cannot achieve complete elimination of malignant tumors [64]. Given the PD-1/PD-L1 ICIs could remove some of the immunosuppressant effect on the central and peripheral tolerance to cancer vaccines, they have been increasingly used in combination with neoantigen-based cancer vaccines to activate the specific T-cells for recognizing the tumor cells and kill them.

Table 2 summarizes the reported clinical trials using neoantigen-based cancer vaccines with PD-L1 inhibitors. Among these studies, the NEO-PV-01 trial (NCT02897765) is the largest one conducted to date. It is an open-label, phase Ib clinical trial of a personalized neoantigen-based vaccine NEO-PV-01 in combination with PD-1 inhibitor nivolumab in patients with advanced melanoma, NSCLC, or bladder cancer [65]. Up to 20 unique peptides (~ 14–35 mer) are selected from each patient’s tumor for manufacture, which took about 12 weeks. The analysis of 82 patients demonstrated that the regimen was safe, with no treatment-related serious adverse events observed. Furthermore, the RECON® (Real-time Epitope Computation for Oncology) pipeline was able to identify high-quality neoantigens for manufacturing and clinical use. These neoantigens in combination with nivolumab stimulated durable neoantigen-specific T cell reactivity that was cytotoxic to tumors in study subjects. De novo neoantigen-specific CD4^+^ and CD8^+^ T cell responses were observed post-vaccination in all patients. The vaccine-induced T cells had both neoantigen-specific response and cytotoxic phenotypes that were capable of trafficking to the tumor and mediated cell death. In addition, antibodies to neoantigens that were not included in the vaccines were also detected post-vaccination samples. This phenomenon is called epitope spreading, which is defined as the diversification of epitope specificity from the initial selected, neoantigen-specific immune response to subdominant and/or cryptic epitopes (neoantigens) on that protein (intramolecular spreading) or other proteins (intermolecular spreading) [66]. This is an important observation, supporting that neoantigen vaccination may increase its efficacy by generating tumor-specific immunity against other driver neoantigens present in the tumor and/or emerged during cancer progression. Furthermore, personal neoantigen peptide vaccines induced T cell responses that persisted over years and broadened the spectrum of tumor-specific cytotoxicity in eight patients with surgically resected stage IIIB/C or IVM1a/b melanoma (NCT01970358) [67]. All patients were alive and six were without evidence of active disease at a median of 4-year follow-up. Personal neoantigen vaccines induced persistent memory T cells and epitope spreading. These data support the feasibility, safety, antigen-specific immunity, and promising early and long-term antitumor activity of this personalized neoantigen-based therapeutic strategy in patients with advanced solid tumors in both metastatic and adjuvant settings. Although the majority of current neoantigen vaccine trials use the peptide delivery platform, mRNA delivery platform has also been investigated with promising results. In the Phase 1 study of the personalized cancer vaccine mRNA-4157 in combination with pembrolizumab for patients with metastatic solid tumors, only low-grade adverse events were observed in all patients. Overall response rate (ORR) was 50% and 14.6% and mPFS was 9.8 months and 2.0 months in patients with Human Papillomavirus (HPV)-negative head and neck squamous cell carcinoma who received mRNA-4157 and pembrolizumab combination and pembrolizumab monotherapy, respectively [68]. With the legend of COVID-19 vaccination, further clinical evaluation of cancer neoantigen vaccination using mRNA delivery platform is highly anticipated in the near future.

**Table 2:** Reported clinical trials of neoantigen-based cancer vaccines with anti-PD1 therapy

NCT numbers (reference)	Cancer vaccine	Phase	Patients	Tumor type	Combination	Outcomes
NCT02529072 [74]	DC vaccines	I	6	Recurrent grade III and grade IV brain tumors	Nivolumab	NA
NCT02981524 [75]	GVAX colon vaccine	II	17	MMR-p advanced colorectal cancer	Pembrolizumab	Failed to meet its primary objective
NCT02879760 [76]	Oncolytic MG1-MAGEA3 with Ad-MAGEA3 vaccine	I–II	16	NSCLC	Pembrolizumab	NA
NCT02515227 [77]	6MHP helper peptide vaccine	I–II	22	Melanoma	Pembrolizumab	NA
NCT02775292 [78]	Peptide-pulsed autologous dendritic cell vaccine	I	1	Solid tumors	Nivolumab	NA
NCT02574533 [79]	Vigil™ autologous vaccine	I	2	Advanced melanoma	Pembrolizumab	NA
NCT02897765 [65]	NEO-PV-01 (personalized neoantigen vaccine)	Ib	82	Melanoma, NSCLC, bladder cancer	Nivolumab	Melanoma: ORR 59%, mPFS 23.5 mos; NSCLC: ORR 39%, mPFS 8.5 mos; bladder cancer: ORR 27%, mPFS 5.8 mos)
NCT01970358 [67]	NeoVax (personalized neoantigen vaccine)	I	8	Melanoma	PD-1 inhibitor	75% of patients were without evidence of active disease at a median of 4-year follow-up
NCT01970358 [68]	mRNA 4257	I	10	HNSCC	Pembrolizumab	ORR 50%; mPFS 9.8 mos

*NSCLC* non-small cell lung cancer; *HNSCC* head and neck squamous cell carcinoma; *mos* months; *mPFS* median progression free survival; *ORR* overall response rate

## Summary and future directions

In summary, recent success of ICIs in tumors with high TMB, which is a measurement of cancer neoantigens and tumor antigenicity, supports the current development of neoantigen-based personalized cancer vaccines for patients with high TMB tumor. Several studies have shown neoantigen vaccines are feasible, safe and have promising clinical activity in patients with high TMB tumors in both metastatic and adjuvant settings. Further studies are needed to define the essential properties for neoantigen and adjuvant with strong tumor-specific immunity, antitumor activity, duration of therapy (memory), adaptability (epitope spreading), and decreased unwanted toxicities. Furthermore, immune modulators may regulate innate and/or adaptive immunity to enhance the effect of cancer vaccination with neoantigens. In addition to cancer treatment, cancer vaccines can be used for primary and secondary prevention [69–72]. For instance, immunization against HPV and hepatitis B virus have been used as primary prevention measures that have prevented one million cancer cases each year [73]. In anticipation of the future research and development, the market for cancer neoantigen vaccines is reported to grow over the next half decade. There has been increased investment from multiple pharmaceutical companies and other healthcare sectors.
